# ANCA Associated Vasculitis and Renal Failure Related to Propylthiouracil and Hyperthyroidism Induced Cholestasis in the Same Case

**DOI:** 10.1155/2014/762528

**Published:** 2014-11-20

**Authors:** Mehmet Tuncay, Emine Kivrakoglu, Itir Yegenaga, Erkan Dervisoglu

**Affiliations:** Department of Internal Medicine, Medical School Kocaeli University, 41380 Kocaeli, Turkey

## Abstract

*Introduction*. Liver involvement due to hyperthyroidism and also ANCA positive vasculitis related renal failure cases were reported separately several times before. However, to our knowledge, these two complications together in the same case had never been observed before. * Case Presentation*. The case of an ANCA positive 71-year-old Caucasian male with renal failure and lung involvement, subclinical hyperthyroidism, and intrahepatic cholestatic jaundice was presented in this paper. After exclusion of all of the other possibilities, cholestatic hepatitis was explained by subclinical hyperthyroidism; renal failure and lung involvement were interpreted as ANCA related vasculitis which might be a side effect of propylthiouracil use. * Conclusion*. The coexistence of these rare conditions in the same patient deserves emphasis and it is worth reporting. This case demonstrates that following the clinical course of the patient is essential after prescribing any medications to see whether any complication occurs or not. If the complications of this case were noticed earlier, it would be possible to treat and to prevent the permanent damages.

## 1. Introduction

It was reported previously that at least one hepatic abnormality was observed in 60.5% of the patients with hyperthyroidism because of hypoxia in hepatocytes [1]. Increased metabolic rate in hyperthyroidism causing extra demand and insufficiency of oxygen demand in hepatocytes was claimed from these abnormalities [2, 3].

Propylthiouracil (PTU) is one of the most common drugs to treat hyperthyroidism which is reported to be responsible for ANCA (anti-neutrophil cytoplasmic autoantibody) positivity in 4% to 46% of cases, and even leading to vasculitis especially in China and Japan [4]. Even though PTU induced ANCA positive vasculitis cases were reported more often in Grave's disease, it also may be related to multinodular toxic goiter [5]. PTU-induced ANCA associated systemic vasculitis (AASV) was reported to be involved with the kidneys, skin, and pulmonary system; furthermore, arthralgia and fever were also observed as the most common symptoms [6]. Kidney involvement was reported to be present from mild to severe degree [7].

Based on these information, this case was found to be interesting and rare to be presented here. The case was interpreted as intrahepatic cholestasis caused by nodular toxic goitre and the drug which was used to treat hyperthyroidism leading to ANCA related vasculitis and renal failure.

## 2. Case Presentation

A 71-year-old Caucasian male was admitted to the outpatient nephrology clinic with the complaints of general malaise, progressive asthenia, weight loss, loss of appetite, jaundice, and decrease in urine volume. On admission, blood pressure was 120/70 mm/Hg; the pulse rate was regular 95/min. He was pale, icteric and dehydrated. On physical examination, no abnormalities were found except hepatosplenomegaly and also multiple nodules in his thyroid glands. He had a history of subclinical hyperthyroidism and hypertension for about 30 years and treated with PTU irregularly, carvedilol (12.5 mg/day), and trimetazidine (60 mg/day). The patient was hospitalized for further investigation.

As it is shown in Tables 1 and 2, he showed normokrom normositer anemia pancytopenia, cholestasis, and subclinical hyperthyroidism in his laboratory examination.

**Figure 1:**
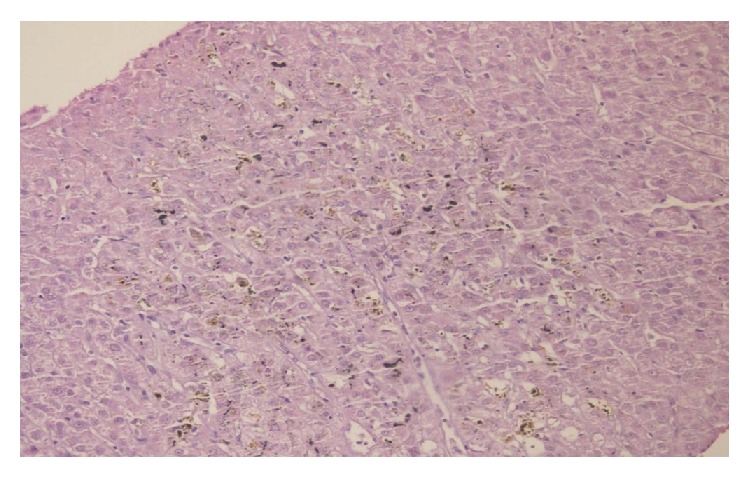
Biopsy material of liver.

Ultrasonographic examination and magnetic resonance imaging (MRI) of abdomen were performed to exclude extrahepatic cholestasis and showed no clues for malignancies but heterogeneity of liver parenchyma; intra- and extrahepatic bile duct sizes were normal; enlargement of liver (170 mm) and spleen (188 mm) were observed; in addition, portal and splenic vein diameters were increased. Heart failure and the other rare causes of intrahepatic cholestasis were also excluded. Pathological examination of liver biopsy showed that there was sparse piece-meal necrosis and inflammatory cell infiltration in the portal areas. Focal ductuli proliferation, pericentral intracytoplasmic bile pigments, bile plugs, and diffuse focal necrosis were also noted (Figure 1). Histopathological findings were interpreted as intrahepatic cholestasis might be related to thyrotoxicosis.

Serological markers, autoantibodies, laboratory findings, and liver biopsy findings helped us to exclude primary biliary cirrhosis, autoimmune hepatitis, Wilson's disease, hemochromatosis, and viral hepatitis.

On his 14th day of hospitalization, tachycardia, elevated blood pressure, tachypnea, agitation, and dysphonia were observed. His renal function was deteriorated and hemodialysis treatment was initiated.

Since PTU and methimazole treatments have been reported to be related with AASV, we decided to perform radioactive iodine (^131^I-10 mCi) treatment on 27th day of admittance for hyperthyroidism. After the RAI treatment, patient became euthyroid but cholestasis was not ameliorated. Ursodeoxycholic acid (1500 mg/day) and rifampicin (600 mg/day) treatments also did not bring any further benefit in point of cholestasis.

Patient was pancytopenic on his admittance and during the follow-up; haematological examination and bone marrow biopsy showed no significant pathology. Serum and urine immune fixation electrophoresis were performed to exclude multiple myeloma, a possible cause of anemia/pancytopenia in the elderly. Moreover, rectal biopsy was negative for the amyloidosis, which might be possible explanation for the hepatosplenomegaly, kidney failure, and pancytopenia as well. And it was concluded that pancytopenia might be related to hypersplenism.

**Figure 2:**
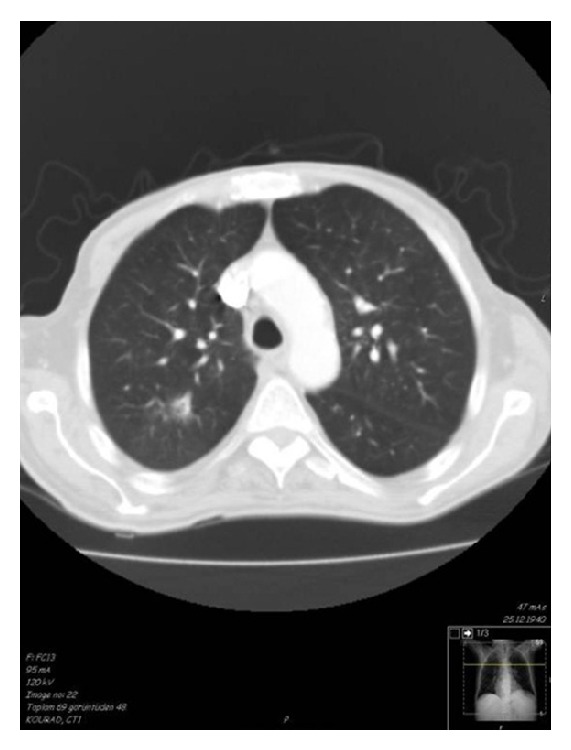
Computerized tomography of lung.

Because the patient had suffered from dysphonia and dyspnea, thorax computerized tomography (CT) was performed and several nodules were discovered in right lung parenchyma (Figure 2); the right apicoposterior one was cavitary; nevertheless, pneumonia, tuberculosis, and malignancies were excluded. These findings supported lung involvement of ANCA positive vasculitis.

After exclusion of all of these possibilities, we concluded that the patient was suffering from hyperthyroidism induced intrahepatic cholestasis. Furthermore, since hyperthyroidism was not taken under control for many years, the changes in the liver became permanent and leading to developed cirrhosis eventually. In addition, we thought that using PTU treatment irregularly to control hyperthyroidism caused ANCA positive vasculitis with renal and lung involvement.

## 3. Discussion

Although during the clinical course of hyperthyroidism mild changes in liver functional tests are often observed, it is reported that in untreated hyperthyroidism pathological changes in liver might progress into focal necrosis and centrilobular intrahepatic cholestasis and eventually cirrhosis can also develop [2]. Nevertheless, subclinical hyperthyroidism induced intrahepatic cholestasis cases like the case presented here are rarely observed [3].

ANCA is usually formed against the serine proteases in monocytes and neutrophils, identified by indirect immunofluorescence. Even though it is not well known, mechanism of PTU-induced AASV is explained as transforming of MPO (myeloperoxidase) into sulfonate by PTU which is highly antigenic and induces autoimmunity. Activated T-lymphocytes stimulate ANCA production by B-lymphocytes and causes vascular injury [8]. It was also reported that higher titration of MPO-ANCA might be the most important risk factor for the development of systemic vasculitis [9]. It was also reported that anti-MPO antibody titration decreases with the immune suppressive therapy or cessation of PTU treatment [10].

In this case, ultrasonographic examination showed small kidneys compatible with chronic kidney failure on admittance; therefore, renal biopsy could not be performed but it was postulated that kidney involvement might be related to vasculitis. However, histopathological examination of kidney in PTU induced that AASV was reported as necrotizing and crescentic pauci-immune glomerulonephritis and total sclerosis [7].

The most common lung involvement in PTU-induced AASV has been reported as diffuse alveolar hemorrhage [11]. But rarely multiple nodules in chest CT were determined similarly in this case [12].

In our case, there were no skin lesions observed but purpuric, erythematous, and necrotic skin lesions were commonly reported in PTU-induced AASV with renal and lung involvement [6] and histopathological examination of these skin biopsy materials showed leukocytoclastic and fibrinoid necrosis [6].

## 4. Conclusion

This case, presented here, carried multiple complications altogether which is interesting and very rare and also we wanted to emphasize that, to follow-up, the results of treatments are very important to see the side effects and complications. If we notice them earlier, it is possible to correct and save the patient from a lot of burden.

## Figures and Tables

**Table 1:** Laboratory findings of the case.

	The day of admission	First dialysis	RAI treatment	Normal values
Hemoglobin (g/dL)	8.97	9.49	7.84	12.2–18.1
Hematocrit (%)	26.3	27.5	23.4	37.7–53.7
Neutrophil (×10³/mL)	1.470	1.930	1.350	4.60–10.2
Thrombocyte (×10³/mL)	135	118	8.4	142–424
Ferritin	558			20–400
Erythrocyte sedimentation rate (mm/h)	82	45	42	0–20
C reactive protein (mg/dL)	5.34	11.4	3	0–0.5
Glucose (mg/dL)	114	116	134	70–105
Urea (mg/dL)	137	254	160	15–55
Creatinine (mg/dL)	2.8	3.87	3.88	0.6–1.53
Uric acid (mg/dL)	9.7	6.9	5.1	2.5–7.7
Arterial pH	7.3	7.1	7.4	7.35–7.45
HCO_3_ (mmol/L)	15.6			22–26
Direct bilirubin (mg/dL)	2.2	8.1	9	0.00–0.50
Aspartate aminotransferase (U/L)	67	34	58	5–34
Alanine aminotransferase (U/L)	58	83	59	0–55
Gamma glutamyl transferase (U/L)	3			9–64
Lactate dehydrogenase (U/L)	127	132	127	125–245
Alkaline Phosphatase (U/L)	1290	1049	932	40–150
T. protein (g/dL)	6.8	6	5.5	6.4–8.3
Calcium (mg/dL)	8.5	8.5	7.9	8.4–10.2
I. phosphorus (mg/dL)	5.3	6.5	3.1	2.7–4.5
Potassium (mEq/L)	5.2	5.1	3.3	3.5–5.1
Chlorine (mEq/L)	110	115	104	98–107
Parathyroid hormone (pg/mL)	56.2			12–88
Free T_3_ (pg/mL)	3.2	8.63	2.12	2.3–4.2
Free T_4_ (ng/dL)	1.25	2.29	0.70	0.74–1.52
Thyroid stimulating hormone (mIU/L)	0.014	0.011	0.547	0.55–4.78

**Table 2:** Immunologic parameters of the case.

	The day of admission	Normal values
CA 125 (OM-MA) (U/mL)	9.58	0.0–30.2
CA 19-9 (GI-MA) (U/mL)	13.1	0.0–35
Carcinoembryonic antigen (ng/mL)	0.18	0.0–0.25
Antithyroidperoxidase (IU/mL)	35.8	0.0–60
Antithyroglobulin (IU/mL)	21.5	0.0–60
Alpha fetoprotein (IU/mL)	1.03	0.5–5.0
Antinuclear antibody	+3 Nuclear membrane	
Antinuclear cytoplasmic antibody (MPO)	+2	
Antinuclear cytoplasmic antibody elastase	+2	
Cytoplasmic antinuclear cytoplasmic antibody	(±)	
Antimitochondrial antibody	(−)	
Anti-liver kidney microsome antibody	(−)	
HBs Ag (S/CO)	<−1.0 (−)	
Anti-HBs (IU/L)	<−10.0 (−)	
Anti-HBcIgG (S/CO)	<−1.0 (−)	
HBe Ag (S/CO)	<−1.0 (−)	
Anti-HBe (S/CO)	>−1.0 (−)	
Anti-HAV IgG (S/CO)	>−1.0 (+)	
Anti-HAV IgM (COI)	<−1.0 (−)	
Anti-HCV (S/CO)	<−1.0 (−)	

HBs Ag: hepatitis B surface antigen, Anti-HBs: anti-hepatitis B surface antibody, Anti-HBe: anti-hepatitis e antigen, Anti-HAV IgG: anti-hepatitis A virus immunoglobulin G, Anti-HAV IgM: anti-hepatitis A virus immunoglobulin M, and Anti-HCV: anti-hepatitis C virus.

## Abbreviations

PTU: Propylthiouracil
ANCA: Anti-neutrophil cytoplasmic autoantibody
AASV: ANCA associated systemic vasculitis
MRI: Magnetic resonance imaging
CT: Computerized Tomography
MPO: Myeloperoxidase.
